# Investigation of truncated replication protein mutant of Canine Circovirus: synergistic interaction with Feline Panleukopenia Virus

**DOI:** 10.3389/fvets.2025.1536913

**Published:** 2025-06-18

**Authors:** Suyao Li, Jialiang Xin, Yi Peng, Haifeng Liu, Ziyao Zhou, Zhijun Zhong, Hualin Fu, Min Yang, Wancheng Li, Ruihu Wu, Guangneng Peng

**Affiliations:** ^1^The Key Laboratory of Animal Disease and Human Health of Sichuan Province, College of Veterinary Medicine, Sichuan Agricultural University, Chengdu, China; ^2^College of Landscape Architecture, Sichuan Agricultural University, Chengdu, China; ^3^Innovative Engineering Research Center of Veterinary Pharmaceutics, Department of Pharmacy, College of Veterinary Medicine, Sichuan Agricultural University, Chengdu, China; ^4^Pet Nutrition and Health Research Center, Chengdu Agricultural College, Chengdu, China; ^5^College of Veterinary Medicine, Sichuan Agricultural University, Chengdu, China

**Keywords:** Canine Circovirus, replication protein, truncated replication protein, Feline Panleukopenia Virus, synergistic effect

## Abstract

**Background:**

Canine Circovirus (CanineCV) is a non-enveloped, single-stranded circular DNA virus in the *Circoviridae* family, known to cause respiratory and diarrheal diseases in dogs. It can also lead to immune suppression, which may worsen symptoms during co-infection. The virus’s Replication (Rep) and Capsid (Cap) proteins play crucial roles in its life cycle. This study explores a novel truncated Rep’ mutant of CanineCV and examines its impact on feline health when co-infected with Feline Panleukopenia Virus (FPV).

**Method:**

We constructed and validated clones and plasmids for CanineCV/ SC49 (which carries the normal Rep gene) and CanineCV/SC50 (which carries the truncated Rep’gene). Virus particles were visualized using transmission electron microscopy (TEM), while quantitative polymerase chain reaction (qPCR) assessed viral load. Additionally, we examined the effects of Rep and Rep’ proteins on cellular viability, their roles in FPV replication, and the host interferon type I (IFN-I) response.

**Results:**

The Rep’ protein significantly enhances the cytotoxicity of CanineCV against the F81 cell line, outperforming the Rep protein in this regard. However, when assessing the proliferation-promoting effects on FPV, both proteins demonstrated positive effects, but Rep exhibited a significantly greater impact than Rep’ Additionally, qPCR analysis revealed that Rep has a stronger inhibitory effect on the expression of IFN-α, IFN-β, MxA, and ISG15 genes compared to Rep’.

**Conclusion:**

This study underscores the dual roles of Canine Circovirus in modulating host cell viability. On one hand, it enhances the replication of co-infecting viruses; on the other hand, it suppresses the host’s antiviral responses. These findings provide valuable insights into the pathogenic mechanisms of Canine Circovirus.

## Introduction

Canine Circovirus (CanineCV) is a small, non-enveloped, single-stranded circular DNA virus that belongs to the *Circoviridae* family and the *Circovirus* genus (1). Since its initial discovery in 2012 (2), this virus has been identified in canines across numerous countries and regions worldwide, including Europe (3, 4), Asia (5–8), and South America (9, 10). CanineCV is associated with respiratory diseases (11) and diarrhea (12–14) in dogs and may potentially lead to immune suppression (15, 16), thereby exacerbating symptoms of co-infection with other pathogens (9).

The replication protein (Rep) and capsid protein (Cap) of Canine Circovirus play crucial roles in the virus’s life cycle. The Rep protein is primarily responsible for viral replication, while the Cap protein forms the viral capsid and is closely linked to the host’s immune response (17, 18). In a previous study, we identified a novel Canine Circovirus featuring a truncated replication protein (Rep’). This isolate, designated SC50, has a truncated ORF1 sequence of 846 nucleotides, encoding only 281 amino acids. Sequence analysis revealed that this truncated Rep’ protein includes a novel C-terminal amino acid sequence: WDQGRPVSTSYFD (19). These mutations in the Rep protein may significantly impact the virus’s biological characteristics, including its virulence, transmissibility, and host range.

CanineCV also demonstrates the potential for cross-species transmission, having been detected in various wild animals, including wolves (13), badgers (20), foxes (21). Notably, recent studies have reported the first evidence of CanineCV infection in cats, with a positive detection rate of 3.42% (22). This raises concerns regarding the potential impact of CanineCV on feline populations, particularly in light of the possibility that the virus may exacerbate pre-existing health conditions in affected cats. In felines, clinical manifestations resulting from co-infection with CanineCV and other pathogens could lead to more severe outcomes, as the immune suppression induced by the virus may impair their ability to combat infections (15, 23, 24). Given the close interactions between cats and dogs, along with potential environmental exposure, these findings highlight the necessity for increased vigilance concerning CanineCV within feline populations (25).

Feline Panleukopenia Virus (FPV) is a highly contagious pathogen that poses a significant threat to the health of felines (26, 27). It primarily targets bone marrow cells, leading to leukopenia and immunosuppression (28, 29). The global prevalence of FPV has been well-documented, highlighting its significant impact on the health and welfare of feline populations (30, 31). Understanding the co-infection dynamics of CanineCV and FPV is essential for elucidating viral interactions, evaluating the effects of co-infection on feline health, and formulating effective prevention and control strategies.

In this study, we successfully rescued CanineCV in F81 cells and characterized the cytotoxic effects of both the full-length Rep protein and its truncated variant, Rep’. Notably, expression of the Rep’ protein not only enhanced FPV replication but also inhibited type I interferon responses. These findings provide important insights into the impact of co-infection with CanineCV and FPV on feline health. By examining these interactions, we aim to establish a scientific foundation for future prevention and control strategies. This research holds significant practical implications for safeguarding companion animal health as well as ensuring public health security.

## Materials and methods

### Cells, viruses and plasmids

F81 cells were cultured in DMEM with 10% heat-inactivated FBS (Gibco), 100 units/mL penicillin, and 100 mg/mL streptomycin at 5% CO₂, 37°C. The pBlueScript II SK (+) was bought from Shanghai Sangong Biotechnology, pCMV-C-mCherry from Shanghai Biyuntian Biotechnology, and pMD19-T from Baozhi Biotechnology in Beijing. CanineCV-DG was from previous research (19). FPV were stored in our laboratory.

### Construction of an infectious clone of CanineCV

Plasmids pBSK-SC49 and the truncated replication protein mutant plasmid of Canine Circovirus pBSK-SC50 were constructed from the full-length sequences OQ910504 and OQ910505 in the NCBI database, and were supplied by Sangyo Bioengineering (Shanghai) Co.

### Construction of eukaryotic expression plasmids

The Rep and Rep’ genes, along with the Cap gene, were amplified using specific primers and subsequently cloned into the pMD-19 T vector to generate the plasmids pMD-19 T-Rep, pMD-19 T-Rep’, and pMD-19 T-Cap. These constructs were then modified with EcoRI and XhoI restriction enzymes, purified, and ligated to produce eukaryotic expression plasmids: pCMV-C-mCherry-Rep, pCMV-C-mCherry-Rep’, and pCMV-C-mCherry-Cap. Detailed information on the primers used is provided in Table 1.

**Table 1:** Primers for eukaryotic expression plasmid construction.

Name	Primers	Sequences (5′ → 3′)
pMD-19 T-Rep	Rep-F	GAATTCATGGCCCAGGCTCAAGTGGATCAG
	Rep-R	CTCGAGGTAGTTATACATATGGGGGAACATAAAG
pMD-19 T-Rep’	Rep’-F	GGAATTCCTTAATCAAAGTATGACGTCGACACAGG
	Rep’-R	CTCGAGATCAAAGTATGACGTCGACACAGG
pMD-19 T-Cap	Cap-F	GAATTCATGCGCGTGCGCAGGCATGCCCGG
	Cap-R	CTCGAGCAATTGGCGGCCAGTCTCATAATCAAAC

### Transmission electron microscopy (TEM)

F81 cells were transfected with pBSK-SC49 and pBSK-SC50 using Lipo3000™ and incubated for a duration of 48 h. Following collection and fixation, the cells underwent a series of procedures including rinsing, osmium fixation, dehydration, acetone treatment, resin infiltration, and staining. The resulting sections were subsequently examined using a Talos L120C transmission electron microscope (TEM).

### Cell viability assay

F81 cells cultured in 96-well plates were transfected with plasmids and incubated for 1 h at 37°C. Following the addition of CCK-8 reagent, absorbance was measured at OD 450 nm. In 6-well plates, cells underwent transfection, were fixed with 4% paraformaldehyde for a minimum of 10 min, washed thoroughly, and subsequently stained with crystal violet for an additional 10 min before being photographed.

### qPCR analysis

For the quantification of viral load, specific qPCR primers, namely JC-Rep for the Rep gene, JC-Cap for the Cap gene, and JC-Rep’ for the Rep’ gene, were deliberately designed. The resulting amplicons were subsequently cloned into the pMD19-T vector, and a standard curve was established by serially diluting purified plasmid DNA from 10^9^ to 10^1^ copies/μL.

To evaluate the expression of interferon-related genes and the alterations in the replication levels of FPV, RNA was extracted and reverse transcribed into complementary DNA (cDNA). The expression levels of IFN-*α*, IFN-*β*, ISG15, and MxA, as well as the replication level of FPV, were quantified relative to GAPDH using the 2^-ΔΔCt^ method. Detailed information regarding the primers can be found in Table 2.

**Table 2:** Primers used for RT-qPCR analysis.

Name	Primers	Sequences (5′ → 3′)
JC-Rep	JC-Rep-F	GTGGATCAGCGCGTCCGAGATAG
	JC-Rep-R	CTTCAGGGCACCCATACGGGTAG
JC-Rep’	JC-Rep’-F	TGGAGGAGGAGGATGCCGTGAAG
	JC-Rep’-R	TCAGGGCACCCATACGGGTAGTC
JC-Cap	JC-Cap-F	ACCATGGGTCTCCGTCATACAAG
	JC-Cap-R	GGCGGCCAGTCTCATAATCAAAC
FPV	FPV-F	CAACCATACCAACTCCAT
	FPV-R	ATTCATCACCTGTTCTTAGTA
IFN-α	IFN-α-F	GCCCTCTTCCTTCTTGGT
	IFN-α-R	GCCTTGTGGGACTGGTCT
IFN-β	IFN-β-F	GTGTGTTTCTTCACCACCGC
	IFN-β-R	GTGTCGCAAGGAGGTTCTCA
ISG15	ISG15-F	TCCTGGTGAGGAACCACAAGGG
	ISG15-R	TTCAGCCAGAACAGGTCGTC
MxA	MxA-F	TCAAGGGCGGAGATGGTT
	MxA-R	AAGGGAGTCGATGAGGTCAA
GAPDH	GAPDH-F	TGGAAAGCCCATCACCATC
	GAPDH-R	ACTCCACAACATACTCAGCACCA

### Statistical analysis

One-way ANOVA was used to determine significant differences (*p <* 0.05) across all experiments. Statistical analyses were performed using the nonparametric test in GraphPad Prism 10.0.

## Results

### The rescue and identification of CanineCV/SC49 and CanineCV/SC50

**Figure 1:**
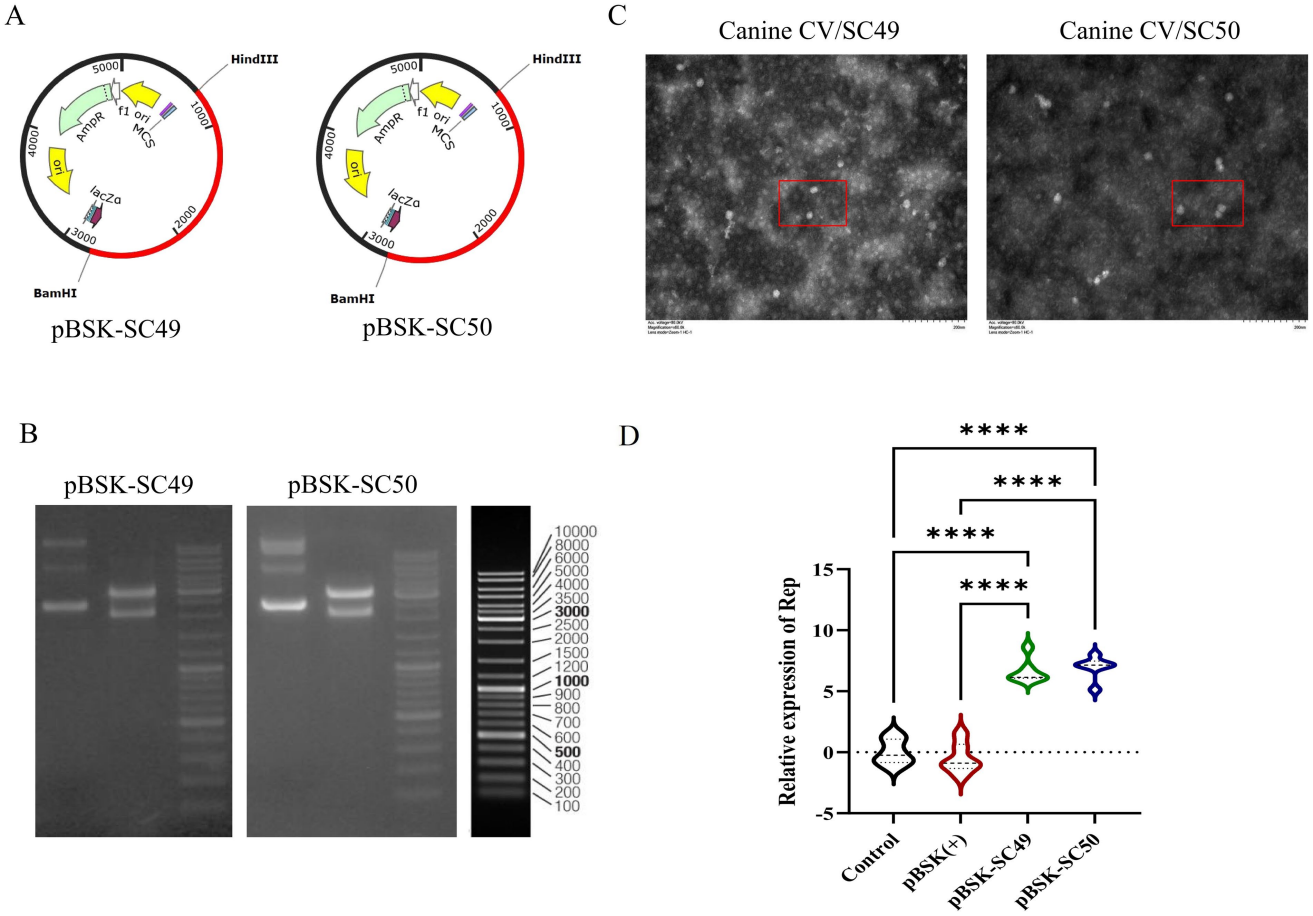
**(A)** Construction of reverse genetic recombinant plasmids pBSK-SC49 and pBSK-SC50. **(B)** The results of the double digestion identification of the recombinant plasmid are presented below. Lane 1 contains the original plasmid, while lane 2 contains the double digestion HindIII/BamHI identification. **(C)** TEM images of transfected pBSK-SC49 and pBSK-SC50 F81 cells. F81 cells were inoculated into 6-well plates and 1 μg of plasmid was transfected into the cells. Forty-eight hours after transfection, cells were collected and processed for observation. CanineCV particles are marked with red boxes. The scale bar represents 500 nm. **(D)** Identification of rescue viruses: qPCR detection of ORF1 expression products. All values represent the mean ± SD. *p < 0.0001.

As illustrated in Figure 1A, the viral cloning plasmids pBSK-SC49 and pBSK-SC50 were successfully constructed. Figure 1B presents the results of the HindIII/BamHI double digestion analysis.

Transmission electron microscopy (TEM) observations revealed a substantial presence of viral particles approximately 20 nm in diameter, along with numerous inclusion bodies in the cytoplasm of infected cells (Figure 1C). After a 48-h transfection period, viral DNA was extracted and analyzed by qPCR to evaluate the expression products of ORF1 (Figure 1D).

Additionally, this study confirmed that both CanineCV/SC49 and CanineCV/SC50 were effectively rescued. However, it was noted that the rescued Canine Circovirus could propagate up to the fourth generation in F81 cells before gradually disappearing. These data are not depicted in this figure.

### CanineCV affects cell viability

**Figure 2:**
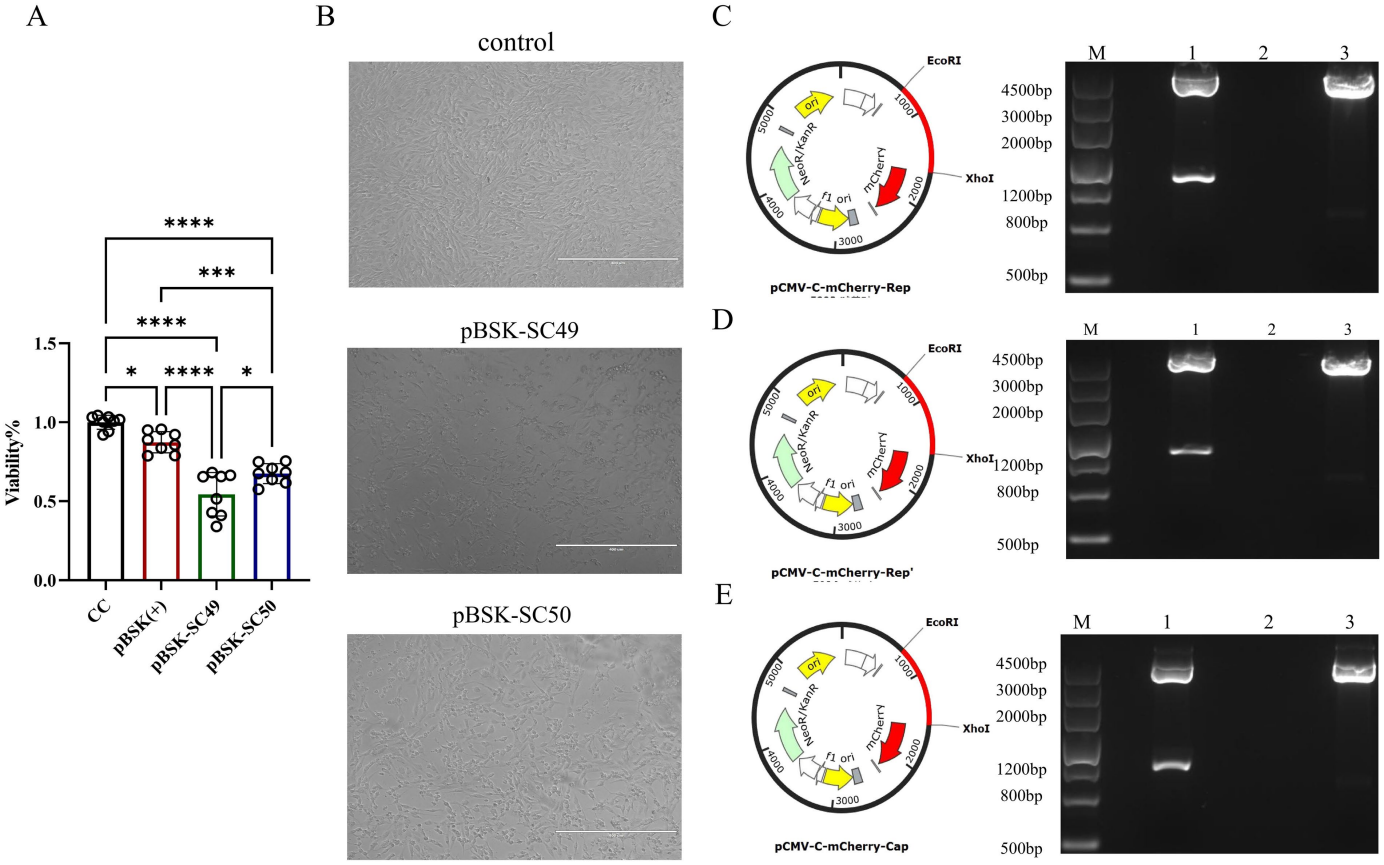
**(A)** The effect of each virus on cell viability over 48 h was examined using the CCK-8 assay. The data illustrated in the figure are presented as the mean ± SD of three independent experiments. The data illustrated in the figure are presented as the mean ± SD of three independent experiments. * *p* < 0.05, *** *p* < 0.001, **** *p* < 0.0001. **(B)** F81 cells were transfected with different virus expression plasmids. The growth state of each group of cells was directly observed under an optical microscope. **(C)** pMCV-C-mCherry-Rep zymography identification. **(D)** pMCV-C-mCherry-Rep’ zymography identification. **(E)** pMCV-C-mCherry-Cap zymography identification.

Both CanineCV/SC49 and CanineCV/SC50 influence cell viability and demonstrate cytotoxic effects. However, CanineCV/SC49 exhibited more pronounced detrimental effects on the cells (Figures 2A,B).

### The Rep’ protein affects cell viability

**Figure 3:**
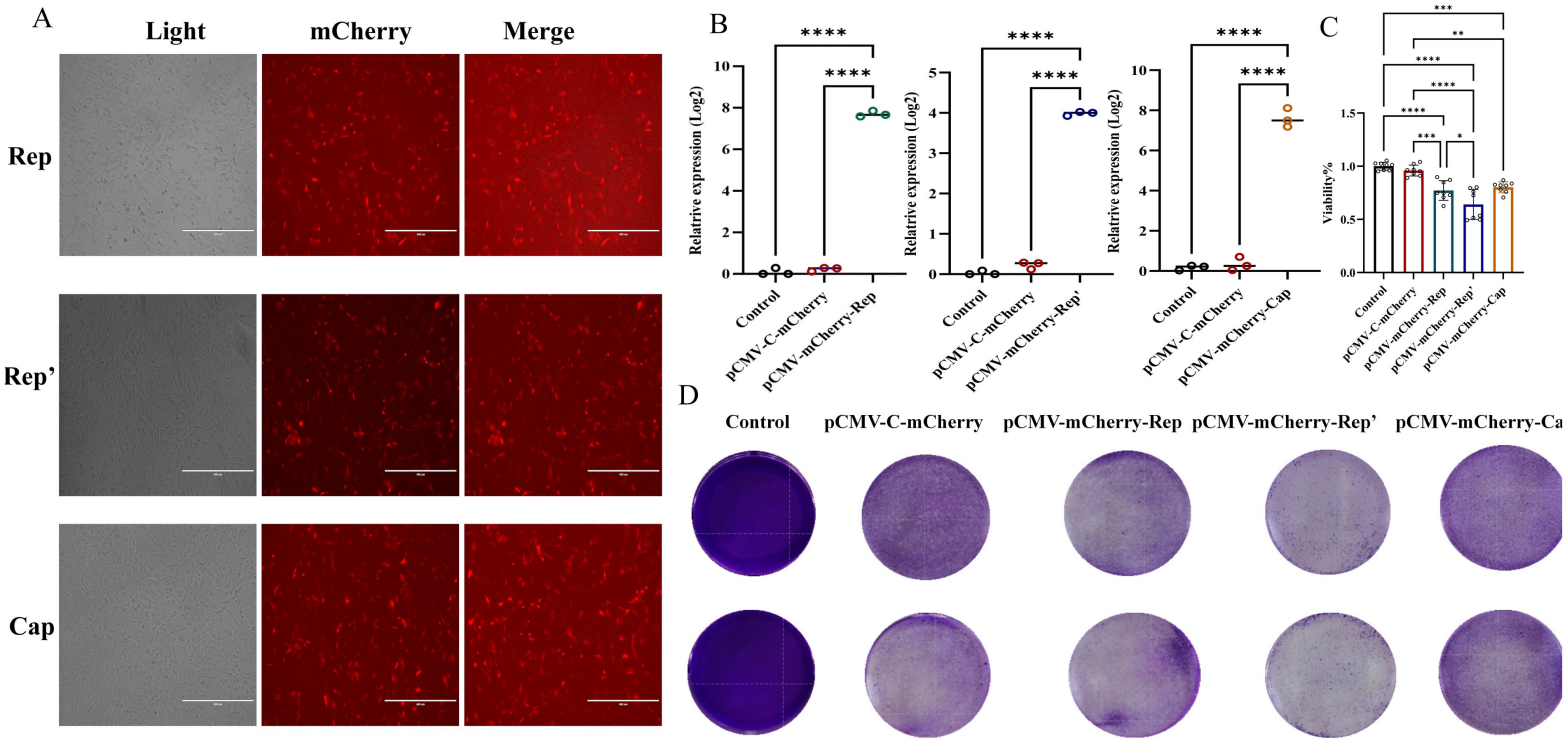
**(A)** Plot of plasmid transfection results in red fluorescent field of view. **(B)** qPCR was employed to detect the expression of the Rep, Rep’, and Cap proteins. **(C)** CCK-8 assays were performed to detect the effect of each viral protein on cell viability at 24 h. The data illustrated in the figure are presented as the mean ± SD of three independent experiments. * *p* < 0.05, *** *p* < 0.001, **** *p* < 0.0001. **(D)** Crystalline violet staining results.

To assess the impact of the Rep’ protein on cell viability, we constructed corresponding eukaryotic expression plasmids: pCMV-C-mCherry-Rep (Figure 2C), pCMV-C-mCherry-Rep’ (Figure 2D), and pCMV-C-mCherry-Cap (Figure 2E). Enzymatic digestion analysis was subsequently performed to confirm their integrity. These plasmids were successfully transfected into F81 cells (Figure 3A), and the expression levels of each protein were assessed independently (Figure 3B).

The CCK-8 assay indicated that the Rep protein led to a significant decline in cell viability, revealing obvious cytotoxicity. Nevertheless, the Rep’ protein, although it also decreased cell viability, exhibited lower cytotoxicity than the Rep protein (Figure 3C).

Crystal violet staining was utilized to assess cell viability across various experimental groups, revealing distinct differences in cell morphology and distribution. The control group and the pCMV-C-mCherry group exhibited high viability, characterized by uniform staining patterns. In contrast, the pCMV-mCherry-Cap group demonstrated similar characteristics with no significant impact on cell viability. In contrast, the pCMV-mCherry-Rep group displayed more transparent areas and a reduction in viability compared to the control. Both the pCMV-mCherry-Rep and pCMV-mCherry-Rep’ groups showed signs of cellular damage or death, with the Rep group exhibiting the lowest level of viability while the control maintained the highest (Figure 3D).

### The Rep’ protein promote FPV replication and immune evasion

**Figure 4:**
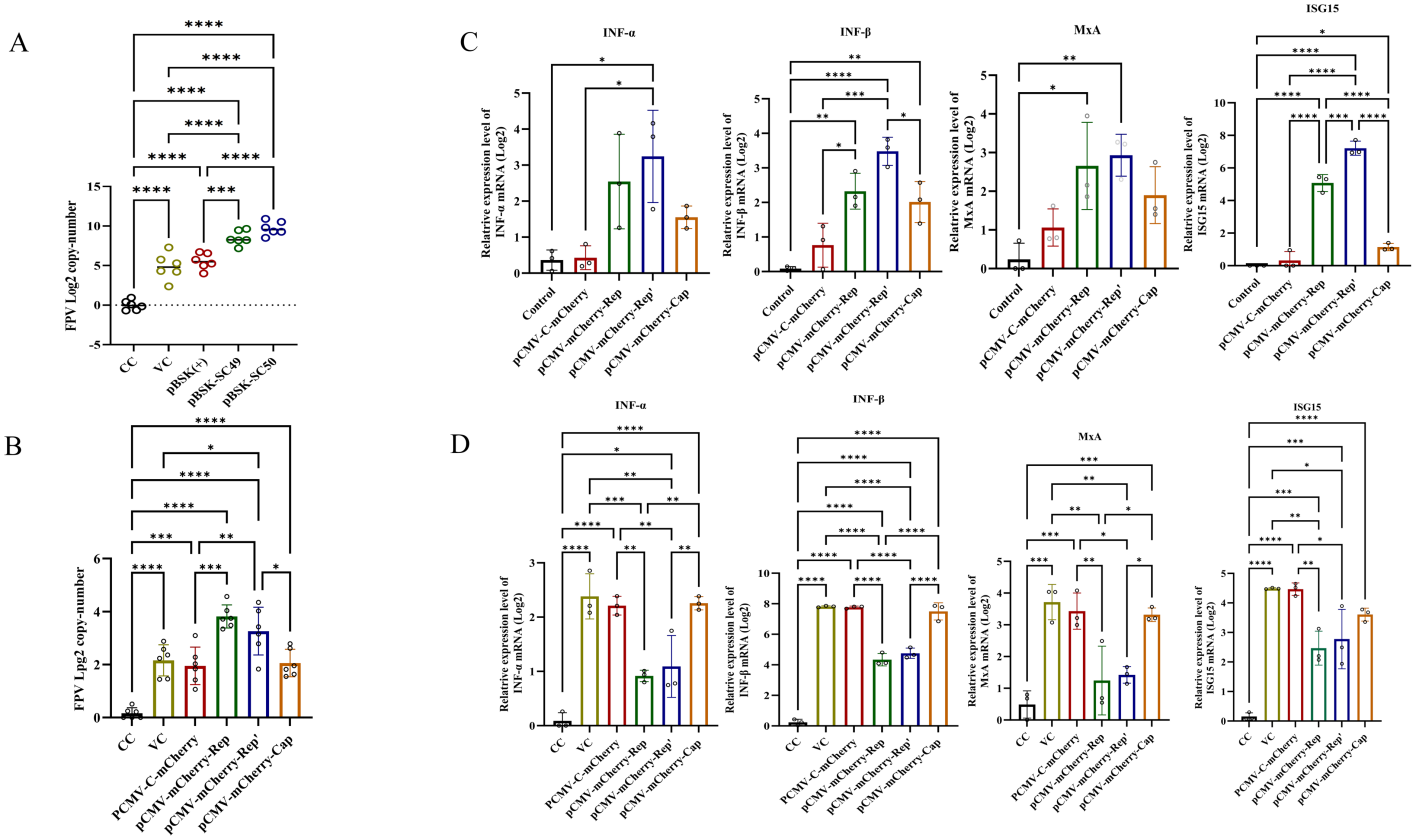
**(A)** F81 cells were transfected with plasmids pBSK, pBSK-SC49, and pBSK-SC50. After 24 h, the cells were inoculated with FPV at an MOI of 1. The cells were harvested 48 h after virus infection, DNA was extracted, and the copy number of FPV was determined by qPCR. **(B)** F81 cells were transfected with Rep, Rep’, and Cap plasmids. Twenty-four hours later, the cells were inoculated with FPV at an MOI of 1. The Rep and Rep’ protein expression groups exhibited higher levels of FPV replication. **(C)** F81 cells were transfected with various plasmids, and total RNA was extracted from the cells. The mRNA levels of IFN-*α*, IFN-*β*, MxA, and ISG15 were then detected using qPCR. **(D)** F81 cells were transfected with different plasmids and subsequently infected with FPV. Total RNA was extracted from these cells, and the expression levels of IFN-*α*, IFN-*β*, MxA, and ISG15 were assessed using qPCR. The data illustrated in the figure are presented as the mean ± SD of three independent experiments. *p < 0.05, ***p < 0.001, ****p < 0.0001.

To investigate whether CanineCV infection enhances FPV replication, F81 cells were transfected with pBSK-SC49 and pBSK-SC50, followed by inoculation with FPV 24 h later. After 48 h of FPV infection, total DNA was extracted and FPV replication was assessed by qPCR. The results indicated that the expression level of FPV following CanineCV/SC49 infection was slightly elevated compared to the control group; However, CanineCV/SC50 infection significantly increased FPV expression, thereby greatly promoting FPV replication (Figure 4A). More interestingly, transfection of pCMV-C-mCherry-Rep or pCMV-C-mCherry-Rep’ was also sufficient to promote FPV replication in cells (Figure 4B).

To investigate the effects of Rep, Rep’, and Cap proteins on the immune response to FPV, we transfected F81 cells with the corresponding plasmids. Twenty-four hours later, these cells were inoculated with FPV and incubated for an additional 48 h to stimulate the cellular IFN-I response. Subsequently, we measured mRNA levels using quantitative PCR (qPCR).

Cells transfected with pCMV-C-mCherry-Rep and pCMV-C-mCherry-Rep’ exhibited significantly lower expression levels of IFN-*α*, IFN-*β*, MxA, and ISG15 compared to those infected solely with FPV. The group transfected with pCMV-C-mCherry-Cap also demonstrated a decrease in expression; however, this reduction was not as pronounced as that observed in the other two plasmid groups (Figure 4C). Notably, in cells infected with FPV, compared to the vector control group (VC group), the expression levels of IFN-α, IFN-β, MxA, and ISG15 were significantly decreased in cells transfected with pCMV-C-mCherry-Rep, pCMV-C-mCherry-Rep’ and pCMV-C-mCherry-Cap (Figure 4D). Overall, our findings indicate that Rep and Rep’ proteins more effectively suppress the immune response to FPV infection than Cap proteins do. This conclusion is supported by the reduced expression levels of IFN-α, IFN-β, ISG15, and MxA in transfected cells when compared to the control group.

## Discussion

This study provides invaluable insights into the interaction between CanineCV and FPV, particularly through the examination of a novel truncated replication protein mutant, Rep’. Our research findings highlight the complex dynamics of viral co-infection and its potential implications for viral pathogenicity as well as host immune responses.

A notable finding of this research is that CanineCV/SC50 exerts a greater deleterious effect on cell viability compared to CanineCV/SC49, likely due to differences in the biological activity of the encoded proteins. Further analysis revealed that the cytotoxicity of the truncated Rep’ protein is significantly higher than that of its normal form, the Rep protein. This suggests that the intact structure of Rep protein is the primary source of cytotoxicity. The underlying mechanism may involve the endonuclease activity of the Rep protein, which cleaves the host genome and triggers the DNA damage response (DDR), leading to cell cycle arrest or apoptosis (32, 33). In addition, existing studies have shown that the mutation at site Y89 of the Rep protein (loss of catalytic activity) deactivates its endonuclease activity and thereby inhibits viral transcription and replication, providing further support for this hypothesis (34). As a truncated variant, the reduce cytotoxicity of the Rep’ protein may be due to competitive binding to host factors or increased of Rep polymerization, thereby increasing its direct damage to host cells (35). The lower survival rate of cells in the Rep’ expression group in this study is consistent with this speculation. However, as the Rep’ protein may not effectively form complexes with certain host proteins, its regulatory effect on cellular signaling pathways requires further investigation. In the future, the specific mechanism of action of the Rep’ protein can be verified by Co-IP or Fluorescence Resonance Energy Transfer (FRET) experiments to further analyze the structure–function relationship of CanineCV proteins and their pathogenic potential in canine and feline hosts.

Both the Rep protein and its truncated variant, Rep’, can enhance FPV replication in F81 cells. However, Rep exhibits lower cytotoxicity compared to the truncated Rep protein. This observation raises a significant question regarding viral adaptability: How do mutations in the Rep protein influence not only virulence but also interactions with co-infecting viruses? The present study suggests that CanineCV may gain an evolutionary advantage in environments where both viruses coexist by promoting FPV replication while mitigating adverse effects on host cell viability. Research has demonstrated that the Rep protein of PCV2 can interact with host proteins (such as HMGCR, c-Myc), thereby activating the p38-MAPK signaling pathway, facilitating the secretion of IL-10 and participating in the rolling circle replication of viral DNA (36). This evidence indirectly supports the hypothesis that the REP protein may promote the expression of DNA polymerase by activating the S phase of the host cell cycle, thus compensating for the deficiency in the replication efficiency of FPV and further exacerbating the disease process. Furthermore, the Rep protein has been shown to be capable of inhibiting the type I interferon (IFN-I) response (37), while the VP2 protein of FPV has been observed to enhance the infectivity of the virus by binding to the transferrin receptor (TFR) of the host cell and concurrently interfering with the host’s immune response through this interaction (38, 39). This synergistic “dual-pathway inhibition” strategy is prevalently observed in multiple viral co-infections and may represent an effective mechanism for enhancing virulence (40).

Additionally, our findings has further demonstrated that both the Rep and Rep’ proteins have the capacity to conspicuously suppress the type I interferon response, a finding that is in accordance with previous studies which have shown that multiple viruses evade immune recognition through analogous mechanisms, thereby enhancing virulence (41, 42). This immune evasion has a significant impact on the host’s ability to resist infection, underscoring the importance of further investigation into the specific signaling pathways targeted by these proteins. Consequently, future studies should center on determining the molecular mechanisms through which the Rep and Rep’ proteins regulate the host immune response, for instance, by inhibiting the JAK–STAT pathway or down-regulating interferon regulatory factors (IRFs), in order to disclose their specific roles in viral pathogenicity and immune evasion (43, 44).

Notwithstanding the findings of this study, it must be acknowledged that the *in vitro* models employed are not without their limitations. While the research outcomes provide insights into viral interactions and cellular responses, they do not fully mirror the complexity of viral pathogenicity *in vivo*. Consequently, conducting in vivo studies to validate our findings is of paramount importance, in order to explore the physiological responses and interactions within living organisms. Combining these studies with mechanistic inquiries will facilitate a more comprehensive understanding of viral dynamics, particularly in the context of co-infections. While the present study concentrated on the CanineCV/SC50 strain, it remains uncertain whether the observed effects are consistent with other strains or other co-infecting viruses. It is therefore recommended that future studies focus on investigating these interactions to determine the universality of our findings and the potential clinical significance of other prevalent strains.

In summary, the present study has demonstrated that the truncated Rep protein of Canine Circovirus (CanineCV) exhibits a distinctive interaction mechanism, thereby augmenting the virulence of other viruses (e.g., FPV) and undermining the key antiviral pathways that regulate the host immune system. The virus has been detected in domestic cats coexisting with dogs, raising significant queries regarding its zoonotic potential (22). In both domestic and shelter environments, the increase in co-infections and severity of diseases among dogs and cats urgently demands precise therapeutic approaches and vigorous public health policies to effectively counter the threat of zoonotic diseases (45). The mounting evidence for a correlation between Canine Circovirus and feline health deterioration underscores the urgency of assessing its influence on public health, particularly in the context of monitoring zoonotic viruses in pet animals (46).

## Data Availability

The original contributions presented in the study are included in the article/supplementary material, further inquiries can be directed to the corresponding author.
